# Understanding them to understand ourselves: The importance of NHP research for translational neuroscience

**DOI:** 10.1016/j.crneur.2022.100049

**Published:** 2022-08-17

**Authors:** Annabella Lear, Stuart N. Baker, Hannah F. Clarke, Angela C. Roberts, Michael C. Schmid, Wendy Jarrett

**Affiliations:** a Understanding Animal Research, Abbey House, 74-76 St John Street, London, EC1M 4DZ, United Kingdom; b Medical School, Newcastle University, Newcastle upon Tyne, NE2 4HH, United Kingdom; c Department of Physiology, Development, and Neuroscience, University of Cambridge, CB2 3DY, Cambridge, United Kingdom; d Behavioural and Clinical Neuroscience Institute, University of Cambridge, CB2 3EB, Cambridge, United Kingdom; e Department of Neuroscience and Movement Science, Faculty of Science and Medicine, University of Fribourg, 1700, Fribourg, Switzerland; f Biosciences Institute, Faculty of Medical Sciences, Newcastle University, NE2 4HH, United Kingdom

**Keywords:** Non-human primate, Animal models, Translational neuroscience, Prefrontal cortex, Visual prostheses, Movement recovery

## Abstract

Studying higher brain function presents fundamental scientific challenges but has great potential for impactful translation to the clinic, supporting the needs of many patients suffering from conditions that relate to neuronal dysfunction. For many key questions relevant to human neurological conditions and clinical interventions, non-human primates (NHPs) remain the only suitable model organism and the only effective way to study the relationship between brain structure and function with the knowledge and tools currently available. Here we present three exemplary studies of current research yielding important findings that are directly translational to human clinical patients but which would be impossible without NHP studies. Our first example shows how studies of the NHP prefrontal cortex are leading to clinically relevant advances and potential new treatments for human neuropsychiatric disorders such as depression and anxiety. Our second example looks at the relevance of NHP research to our understanding of visual pathways and the visual cortex, leading to visual prostheses that offer treatments for otherwise blind patients. Finally, we consider recent advances in treatments leading to improved recovery of movement and motor control in stroke patients, resulting from our improved understanding of brain stem parallel pathways involved in movement in NHPs. The case for using NHPs in neuroscience research, and the direct benefits to human patients, is strong but has rarely been set out directly. This paper reviews three very different areas of neuroscience research, expressly highlighting the unique insights offered to each by NHP studies and their direct applicability to human clinical conditions.

## Highlights

• Primates have shared neurological features that are not found in other animals.
• Some clinically-relevant neuroscience research can only be done using monkeys.
• A human-like prefrontal cortex makes monkeys suitable for studying anxiety.
• Studies in monkeys have led to development of visual prostheses.
• Research in monkeys helped show how the brain controls movement after stroke.

## Graphical abstract

**Figure:**
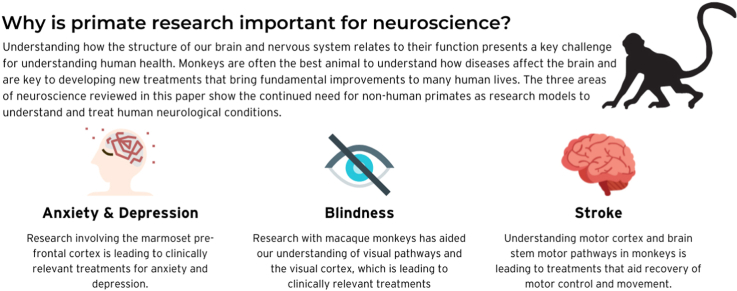

## 1 Introduction

Understanding how the human brain works has challenged researchers for centuries, as has the need to ascertain what goes wrong in the brain in neurological disorders and mental health conditions. The brain’s complexities and capacity to change in response to its environment mean that understanding it remains a fundamental scientific challenge, and our limited understanding about how the brain functions and goes awry affects both social and medical progress. Neurological disorders continue to be a leading cause of disability worldwide and their contribution to the overall burden from all health conditions is increasing (GBD, 2016 Neurology Collaborators, 2019). Examining just one mental health condition demonstrates the scale of the problem, with over 264 million people across the globe currently living with depression (GBD, 2017 Disease and Injury Incidence and Prevalence Collaborators, 2018).

Much progress can be made in understanding the human brain using modern non-invasive methods. For example, transcranial magnetic brain stimulation (TMS) can be used to activate circuits through the scalp; electroencephalogram (EEG) and functional magnetic resonance imaging (fMRI) can elucidate how neural systems are activated by a given task or stimulus. However, these approaches have fundamental physical limits on their spatial and temporal resolution which prevent study of computation at the cellular level. Only more invasive approaches can provide data at the high resolution which is often needed to address important scientific questions. In addition, direct alterations to specific brain areas are a powerful way of revealing causal impacts on function. None of these approaches can be ethically applied to humans. While much brain research is possible using rats and other species, only primates have a human-like complexity to their cortex. Thus, many of the most important social problems that concern the brain cannot be studied in other animals, making research using non-human primates such as macaques and marmosets essential (Homberg et al., 2021). Non-human primates share many neurological processes and functions with humans, developing many of the same diseases and dysfunctions. These include an evolved visual system supporting trichromatic high-resolution visual exploration (Barton, 1998), an expanded association cortex, including prefrontal association cortex, enabling high levels of cognitive control (Wise, 2008, Smaers J et al., 2008), (Roberts and Clarke (2019) and a primate-specific motor system with direct pyramidal tract connections enabling manual dexterity (Lemon and Griffiths, 2005), as outlined in further detail below. The case for using primates in neuroscience is thus strong, but it is rarely set out directly. It may be possible in the future for computer programs, organs on chips, or brain organoids to reduce the need for non-human primates in research, but those technologies cannot yet replace a living animal in neuroscientific experiments. For now, if progress is to be made towards finding cures, research using non-human primates must continue.

In this article, we offer three examples of different ways in which non-human primates are being used to help understand cognition, behaviour, and how illness and brain damage might be treated. As with deep brain stimulation for Parkinson’s disease and other conditions (Krauss et al., 2021) and cochlear implants for deafness (Roche and Hansen, 2015), we hope that the results of the work described below will directly benefit patients in the near future.

All experimental work carried out by authors of this paper and described here was carried out in accordance with the UK Animals (Scientific Procedures) Act, 1986 and associated guidelines, EU Directive 2010/63/EU for animal experiments, or the National Research Council's Guide for the Care and Use of Laboratory Animals, and complied with the ARRIVE guidelines for publication standards.

## 2 Understanding psychiatric disorders through studies of the prefrontal cortex

The prefrontal cortex (PFC), located in the anterior frontal lobes of the brain, is strongly associated with cognitive and emotional symptoms of many human neuropsychiatric disorders. Its normal function, however, remains only partially understood and its dysfunction is opaque. Neuropsychiatric disorders that involve the PFC, including depression, anxiety, schizophrenia, and drug abuse, bring not only a profoundly reduced quality of life for patients and their families, but a considerable economic burden to society. A lack of understanding of the PFC severely restricts the development of new neuropsychiatric treatments, and the failure rate for experimental drugs in this field is high (Hyman, 2012) (Gribkoff and Kaczmarek, 2017).

The PFC contains huge numbers of neurons and support (glial) cells, which communicate in complex ways, so that studying isolated cells provides only limited information about prefrontal operation. Computational models and cerebral organoids are still in their infancy, lacking the sophistication necessary to reveal prefrontal function and dysfunction, and they may never mimic the complexity of the human PFC. Human studies of the PFC are limited by researchers’ inability to manipulate the human brain directly. Most studies of the human brain correlate behaviour with proxy measures of brain function such as cerebral blood flow, and this limits their capacity to establish causality by manipulating function.

Because of these limitations, many neuroscience studies use rodents to determine causality, translating the results to humans as far as possible. However, when studying PFC function this approach is extremely limited, because there are profound anatomical differences between the human and rodent PFC. The neocortex occupies 80% of the human brain but only 28% of the rat’s. This primate-specific neocortical expansion means that many primate PFC brain areas are either unidentified in, or structurally and functionally distinct from, rodents.

Experimental studies in non-human primates allow researchers to bridge this translational gap (Roberts, 2020) and make sense of data from human studies. Some studies use the common marmoset, a small New World monkey with a PFC quite similar to the human. Brain activity within individual marmoset PFC regions can be manipulated temporarily, for example by blocking or boosting the action of a neurotransmitter by injecting drugs through implanted cannulae, or permanently, by destroying specific neuronal subtypes under anaesthesia-although it should be noted that the former more closely mimics what is seen in patients. The effects of these manipulations on the marmosets’ behaviour are measured when they are freely moving around their colonies and through their ability to perform touchscreen-based tasks for desirable food rewards. Crucially, the monkeys, unlike rodents, can perform sophisticated behavioural tasks that are similar to those used to test and diagnose patients in the clinic.

One example is in studies of cognitive inflexibility, a difficulty in modifying behaviour in response to rapidly changing circumstances in the outside world. This is a common deficit in many psychiatric disorders and can slow recovery. Research in marmosets has now revealed distinct forms of cognitive flexibility mediated by distinct regions of PFC and associated neural circuitry (Dias et al., 1996). These different forms of flexibility include reversal learning and attentional set-shifting. Reversal learning is the ability to switch responses from a previously rewarded stimulus to a previously unrewarded stimulus, and attentional set-shifting is where attention needs to shift from a previously relevant category of stimuli (e.g. colour), to a previously irrelevant category (e.g. shape). These abilities are differentially modulated by the neurotransmitters dopamine and serotonin, which are targets that many drugs use to treat psychiatric disorders. Numerous studies in marmosets revealed localised and differential dopaminergic and serotonergic actions within distinct prefrontal circuitry (Crofts et al., 2021) (Clarke et al., 2014) (Wallis et al., 2019) (Clarke et al., 2004) (Clarke et al., 2005) (Clarke et al., 2008) (Clarke et al., 2011). These findings in marmosets led directly to the identification of different forms of inflexibility in distinct psychiatric disorders (reviewed in Oikonomidis et al., 2017), opening up the potential for more effective targeted treatment.

In other examples, the use of temporary inactivation, overactivation, and modulation of PFC regions has revealed the brain circuits that control choosing, and responding to, emotional stimuli, generating findings relevant to human depression and anxiety (Clarke et al., 2015) (Alexander et al., 2019) (Wallis et al., 2019) (Agustín-Pavón et al., 2012). Studies demonstrate that innate anxiety levels in marmosets are associated with prefrontal dysfunction, and that an anxious phenotype can be induced by dysregulating distinct prefrontal areas. This has revealed biologically distinct forms of anxiety that should help to stratify disorders and create individualised treatments (Roberts, 2020). Identifying PFC subregions that regulate the response to threat (related to anxiety), reward (related to anhedonia, or lack of pleasure, a core symptom of depression) and cardiovascular function (Wallis et al., 2017) (Zeredo et al., 2019) (Alexander et al., 2020), has also explained why depression and anxiety are often co-morbid and why depression and cardiovascular dysfunction often co-occur in humans (Roberts and Carke, 2019). Studying how antidepressants affect emotional behaviour in marmosets with different gene variants is providing insights into the varied response to antidepressants among patients (Santangelo et al., 2016). Moreover, differentiating the effectiveness of distinct forms of antidepressants in ameliorating distinct psychiatric symptoms, e.g. heightened anxiety and blunted reward responsivity, in some cases induced by the same PFC manipulation, e.g. subcallosal cingulate area 25 (Alexander et al., 2019, 2020), is providing important new insights into individualised treatment strategies.

## 3 The need for NHPs to develop a visual prosthesis for the blind

Designing a prosthesis to recover vision for the estimated 40 million blind people worldwide (Bourne et al., 2013) has been a long-standing aim of biomedicine. The aim of prosthetic development is to induce meaningful visual perception in otherwise blind patients by stimulating intact parts of their visual system. Over several decades, prosthetics have been developed to interface with the visual system at different levels. Treatment of retinitis pigmentosa, a condition of inherited photoreceptor loss leading to blindness, targets the retina and optic nerve. However, more severe forms of blindness affecting the optic nerve require stimulation of visual areas of the brain (Ghezzi, 2015) (Lewis et al., 2015). In recent visual prosthesis development, animal models have been critical, particularly the macaque, an Old World monkey with a highly evolved visual system. Like us, macaques have forward-facing eyes, foveal high-resolution and trichromatic vision, and human-like visual brain organization (Kaas and Collins, 2003).

Macaque studies have allowed the neural computations and cognitive processes carried out at different levels of the visual system to be fully investigated, revealing relationships between structure and function that are conserved in humans. For example, delineating how the microarchitecture of the visual cortex integrates feedforward and feedback inputs, to process and select information from the two eyes about oriented edges, colour and motion for visual awareness (Callaway, 2005) (Hubel and Wiesel, 1977) (Leopold, 2012), has provided crucial information for designing meaningful stimulation of the visual cortex (Roelfsema and Treue, 2014) (Schiller and Tehovnik, 2008). Studies have also contributed to understanding of the plasticity in neural wiring following retinal disease, which can impose limits on prosthetic approaches (Gilbert and Li, 2012) (Wandell and Smirnakis, 2009). As monkeys cannot communicate verbally, carefully designed testing paradigms have allowed researchers to consistently interpret the monkeys' perceptual experience and test the prosthesis’s effectiveness (Schiller et al., 2011). Some promising recent approaches have successfully generated vision in monkeys and then in humans by electrically stimulating their visual cortexes using hundreds of electrodes (Beauchamp et al., 2020) (Chen et al., 2020). In the studies with monkeys, researchers used their knowledge of cortical topography to project letters into patterned electrical stimulation of the visual cortex, which the monkeys then successfully reported through eye movements (Chen et al., 2020). There are now full clinical trials underway to extend this promising approach of visual cortical prosthetic stimulation (NIH ClinicalTrials.gov, 2017).

In parallel to these successes in achieving prosthetic vision with electrical stimulation, increasing progress is being made with optogenetic stimulation. Following the invention of optogenetics for neuroscience (the use of gene therapy to make specific neurons susceptible to light stimulation) (Boyden et al., 2005), the method has increasingly been adapted for applications in the primate brain (Tremblay et al., 2020). Recent studies of the primate visual system have demonstrated that optogenetic stimulation can adequately drive visual brain circuits and generate visual perception that is similar to that generated by electrical stimulation (Andrei et al., 2019) (Jazayeri et al., 2012) (Klein et al., 2016) (Ortiz-Rios et al., 2021). In addition to this targeting of visual brain circuits, a group of scientists has recently succeeded in translating optogenetic stimulation of retinal ganglion cells, initially carried out in mice, into similar experiments in monkeys. Their work is now showing positive results in an ongoing clinical trial (NIH ClinicalTrials.gov, 2017a, NIH ClinicalTrials.gov, 2017b) (Busskamp et al., 2010) (Gauvain et al., 2021) (Sahel et al., 2021). The patient in the initial case study benefited from wearing goggles that translated camera images into patterned optogenetic stimulation of the eye, enabling him to successfully detect certain objects in his visual field that he would have otherwise been blind to due to retinitis pigmentosa (Sahel et al., 2021).

## 4 Brainstem contributions to stroke recovery

Compared with lower mammals, primates have evolved new circuits for the control of movement which are of critical importance to research into movement recovery. Rodents have two motor cortical regions (Rouiller et al., 1993), primates at least eight (Rizzolatti et al., 1998) (Rathelot and Strick, 2009). While rodents have only indirect pathways between the corticospinal tract and motoneurons, in primates there are monosynaptic cortico-motoneuronal connections (Lemon, 2008). These new circuits underpin the unique motor abilities of primates, such as fine finger movements and a complex range of hand grasps. Much important research has focussed on these newer cortical systems, but primates also retain an evolutionary heritage of subcortical systems, similar to those in lower species. Around 15 years ago, the Baker laboratory began studying the role these phylogenetically older circuits might play in primate movement. Working in monkeys, they found that one primitive descending pathway, the reticulospinal tract, made connections to spinal motoneurons and interneurons involved in hand function (Riddle et al., 2009) (Riddle and Baker, 2010). Reticular formation cells in the brainstem modulated their firing during precise finger movements just as much as corticospinal cells in the motor cortex (Soteropoulos et al., 2012), which showed that control of the primate hand is shared between primitive (reticulospinal) and newer (corticospinal) pathways, each contributing different aspects to the overall function (Zaaimi et al., 2018).

Understanding that there are multiple parallel pathways to control the hand is important when considering the effects of damage. A stroke is caused by ischaemia or haemorrhage in the brain, and usually affects the primary motor cortex, leading to weakness or paralysis. Damaged brain tissue cannot be replaced, but the highly distributed nature of the primate motor system gives options for recovery of function using spared tissue. This may involve surviving cortical areas, such as the supplementary motor area, which has its own corticospinal connections (McNeal et al., 2010). Once it was realised that multiple parallel descending tracts are involved in hand function, it became clear that recovery could also involve using reticulospinal systems to replace lost corticospinal inputs to motoneurons. Zaaimi et al. (2012) tested this hypothesis by making unilateral corticospinal lesions in macaque monkeys, allowing them to recover, and then assessing reticulospinal connectivity. They found that while the reticulospinal tract did indeed strengthen after corticospinal lesions, strengthening occurred in flexor muscles, but not extensors. This likely explains why human stroke patients often recover good finger and wrist flexion but remain permanently weak in extension (Kamper et al., 2003). They have a strong grasp, but persistent problems with hand opening to release an object.

As researchers considered ways of modulating the reticulospinal tract to improve recovery, a serendipitous finding proved critical. Fisher et al. (2012) performed a series of recordings from monkey brainstem, with the original aim of testing whether non-invasive transcranial magnetic brain stimulation (TMS) delivered over the motor cortex could activate reticulospinal cells indirectly via cortico-reticular connections. This indeed proved to be the case, but unexpectedly reticulospinal cells were also found to respond powerfully to the loud click sound made when the TMS machine discharged. This suggested a way to activate these deep cells non-invasively using clicks. Foysal et al. (2016) exploited this effect by developing a wearable device for human use, which paired clicks with electrical stimulus to a muscle. Consistent stimulus pairing over hours with this device seemed to induce long-term changes consistent with spike-timing dependent plasticity in reticulospinal cells, a pathway supported by more recent work (Germann and Baker, 2021). A clinical trial of this device in stroke patients showed a long-lasting benefit on hand function (Choudhury et al., 2020).

## 5 Discussion

There are many methods used to investigate the human brain, but studying living animals with nervous systems similar to our own can sometimes be the only way to answer key scientific questions about brain structure and function. Research on animal nervous systems, compared and translated to equivalent structures in humans, has provided fundamental insights into how the nervous system works, leading to the development of treatments for conditions such as Parkinson’s and Alzheimer’s diseases. Yet many people are still living with neurological disorders for which we do not yet have meaningful treatments, let alone cures. Every item of research carried out tells a different story and adds another piece to the immensely complex puzzle of how our brains work. Here, we have considered three examples of the critical role played by non-human primates in advancing different aspects of neuroscience with direct relevance to human patients.

Using small numbers of primates under strict regulatory controls is providing critical neurobiological understanding of disabling disorders mediated by the PFC, such as anxiety and depression. By revealing the multiple and overlapping contributions of the PFC to aspects of cognitive function, such as flexibility and anxiety, this research is providing insight into the complex interplay between cognition and emotion and how, for example, the symptom of anxiety, can be a product of dysregulation in distinct PFC circuits. This, in part, explains the marked individual differences in treatment response, and consequently these findings are of enormous importance for guiding the development of new individualised treatment strategies, whether they be psychological, pharmacological or surgical.

In the field of visual prostheses, knowledge of the primate visual system has allowed the development of devices that enable human patients with complex blindness to perceive objects visually. While individual human case studies, such as those described here, are encouraging, they illustrate how much basic research work still needs to be carried out if we are to further our understanding of visual system function and advance prosthetic technologies.

Our third example examined the direct benefits to stroke patients gained from studying the control of movement in macaques: work which, like the other examples presented here, could not be done in other mammals which simply do not possess the appropriate neural pathways. Such curiosity-driven fundamental research into the control of movement, has been driven by the human need to understand the world around us, and the substantial differences between primates and other mammals. However, work focussed on understanding movement has nonetheless led directly to significant clinical benefits.

In all the areas we have considered, continued research will help us answer important questions about how the structure of the brain relates to our thoughts, movements, senses and behaviours. While all the methods described above are useful, they each give complementary information, reliant on research from other areas, using other species and other methods for context. Each study serves to provide a single piece of research to build a more complete picture as, for example, no other method can give the spatial and temporal specificity of single cell electrophysiology and neuroanatomy, or the clarity about causality that can be inferred using experimental lesions.

None of the research presented in this paper would have been possible using current non-animal replacement technologies, which cannot replicate the complexities and interconnectedness of the brain’s pathways. All of these areas of research, however, rely on data obtained from monkeys to complement non-invasive methods in humans, and provide clear examples of the progress that can be made at the interface of methods.

As we gain better understanding of these neurological circuits and pathways, they will undoubtedly reveal greater translational benefits for patients living with disabilities, which move beyond the examples of new and developing therapies highlighted in this article. Testing for these future research avenues is also likely to require a joined-up approach, using data from human and non-human primate studies, supplemented by work in rodents where appropriate, to provide better access to new and effective treatments and improve quality of life.

## CRediT authorship contribution statement

**Annabella Lear:** Writing – original draft, Writing – review & editing. **Stuart N. Baker:** Writing – original draft. **Hannah F. Clarke:** Writing – original draft. **Angela C. Roberts:** Writing – original draft. **Michael C. Schmid:** Writing – original draft. **Wendy Jarrett:** Writing – original draft.

## Declaration of competing interest

The authors declare that they have no known competing financial interests or personal relationships that could have appeared to influence the work reported in this paper.
